# A study of medication errors during the prescription stage in the pediatric critical care services of a secondary-tertiary level public hospital

**DOI:** 10.1186/s12887-020-02442-w

**Published:** 2020-12-05

**Authors:** Lorena Michele Brennan-Bourdon, Alan O. Vázquez-Alvarez, Jahaira Gallegos-Llamas, Manuel Koninckx-Cañada, José Luis Marco-Garbayo, Selene G. Huerta-Olvera

**Affiliations:** 1 Comisión para la Protección contra Riesgos Sanitarios del Estado de Jalisco (COPRISJAL), Guadalajara, Jalisco Mexico; 2 Instituto de Terapéutica Experimental y Clínica (INTEC). Departamento de Fisiología. Centro Universitario de Ciencias de la Salud, Universidad de Guadalajara, Guadalajara, Jalisco Mexico; 3 Egresada de la Licenciatura en Químico Fármaco Biólogo, Centro Universitario de la Ciénega, Universidad de Guadalajara, Guadalajara, Jalisco Mexico; 4 Servicio de Farmacia, Hospital Francesc de Borja, Gandía, Valencia Spain; 5 Departamento de Ciencias Médicas y de la Vida. Centro Universitario de la Ciénega. Universidad de Guadalajara, Guadalajara, Jalisco Mexico

**Keywords:** Medication errors, Prescription error, Dispensing error, Pediatric emergency, Pediatric critical care

## Abstract

**Background:**

Medication Errors (MEs) are considered the most common type of error in pediatric critical care services. Moreover, the ME rate in pediatric patients is up to three times higher than the rate for adults. Nevertheless, information in pediatric population is still limited, particularly in emergency/critical care practice. The purpose of this study was to describe and analyze MEs in the pediatric critical care services during the prescription stage in a Mexican secondary-tertiary level public hospital.

**Methods:**

A cross-sectional study to detect MEs was performed in all pediatric critical care services [pediatric emergency care (PEC), pediatric intensive care unit (PICU), neonatal intensive care unit (NICU), and neonatal intermediate care unit (NIMCU)] of a public teaching hospital. A pharmacist identified MEs by direct observation as the error detection method and MEs were classified according to the updated classification for medication errors by the Ruíz-Jarabo 2000 working group. Thereafter, these were subclassified in clinically relevant MEs.

**Results:**

In 2347 prescriptions from 301 patients from all critical care services, a total of 1252 potential MEs (72%) were identified, and of these 379 were considered as clinically relevant due to their potential harm. The area with the highest number of MEs was PICU (*n* = 867). The ME rate was > 50% in all pediatric critical care services and PICU had the highest ME/patient index (13.1). The most frequent MEs were use of abbreviations (50.9%) and wrong speed rate of administration (11.4%), and only 11.7% of the total drugs were considered as ideal medication orders.

**Conclusion:**

Clinically relevant medication errors can range from mild skin reactions to severe conditions that place the patient’s life at risk. The role of pharmacists through the detection and timely intervention during the prescription and other stages of the medication use process can improve drug safety in pediatric critical care services.

## Background

Medication errors (MEs) are considered the single most preventable cause of patient injury derived from human factors [1]. MEs are defined by the National Coordination Council for Medication Error Reporting and Prevention (NCC MERP) as “any preventable event that may cause or lead to inappropriate medication use or patient harm while the medication is in the control of the health care professional, patient, or consumer” [2]. These can occur in the following stages of the medication use process: prescribing, transcribing, dispensing, administration, or monitoring [3]. Evidence shows that MEs occur most frequently during the prescription and administration stages [4–8]. A higher incidence of MEs has also been reported in emergency and intensive care services [9–12]. Children, including neonates and premature infants treated in these settings, are more prone to MEs because of high-stake situations that pertain to critically ill children, particularly weight-based dosing miscalculations and timely administration of medications [13, 14]. Moreover, the medication error rate in children has been reported up to three times higher than in adult patients [15].

Nevertheless, while the incidence and classification of MEs has been well studied in adult hospitalized patients [4, 7, 10–12], clear information in pediatric population is scarce. In Latin American hospitals, including Mexico, most hospitals are public and have an increased demand for healthcare services, however, the active participation of pharmacists involved in patient care is very restricted [16, 17].

To the best of our knowledge, studies focused on MEs in pediatric patients admitted to the emergency and critical care units within Mexican public hospitals are limited. Therefore, the objective of this study was to describe MEs during the prescription stage in the pediatric critical care services of a Mexican secondary-tertiary level public hospital.

## Methods

### Study design

A cross-sectional study was performed in a secondary-tertiary public teaching hospital in western Mexico from March 2017 to November 2018 in the following pediatric critical care services: pediatric emergency care (PEC), neonatal intensive care unit (NICU), neonatal intermediate care unit (NIMCU), and pediatric intensive care unit (PICU). The study was approved by the Hospital’s Bioethics and Research Committees.

### Detection system and analysis of MEs

The inclusion criteria were patients in the age range from newborns to 6 months for NICU and NIMCU, and for PEC and PICU, from 1 month to 18 years of age with at least one prescribed medication order during their hospital stay.

The classification of MEs was based on the updated version by the Ruíz-Jarabo working group [18]. During the evaluation period in the different critical care areas, a member of the research team (J.G.L.) collected and analyzed the medication orders from each critical care service using the direct observation technique to detect MEs [19, 20]. In this study, all medication orders [handwritten (legible or illegible), digital, or combined] include all prescribed drugs and are issued by attending physicians. A single medication order was analyzed per patient and each listed drug within the medication order was considered an individual prescription (drug name, dose, route of administration, speed rate of administration, and frequency of administration). Those patients without MEs in their medication orders were considered as “ideal medication orders”. Handwritten medication orders are the norm and prescriptions that include abbreviations or the brand name of the drug instead of the active ingredient were considered outside the norm, and thus, classified as medication errors. Internationally recognized abbreviations were excluded. All detected MEs during the prescription stage were classified according to the medication error taxonomy defined by the Ruíz-Jarabo working group. Subsequently, MEs were analyzed to identify only those clinically relevant due to their potential for patient harm. These variables included wrong speed of administration, misinterpretation/mistaken drug units, and abbreviations for concentrated electrolytes and anticonvulsants. Abbreviations for saline and glucose/dextrose solutions were not considered.

The characteristics of MEs described in this study include number of orders, number of prescriptions, number of prescriptions per order (P/O), type of medication orders, number of illegible medication orders, and amendments. The following variables were evaluated: total number of potential medication errors and clinically relevant medication errors, prescription error rate ([total number of incorrect prescriptions/number of prescribed drugs] × 100), ME/patient index (number of identified medication errors/total number of patients), type of medication error, percentage of patients with ideal medication orders, and prescription with ideal drugs.

The estimated number of medication orders for the four selected areas was previously established by a sample size calculation. The sample size of 301 hospitalized pediatric patients was determined by the formula described by Mendelhall et al. [21] considering the number of admissions in each critical care service and the average of MEs from previous studies [22–24].

### Statistical analysis

Quantitative data were expressed as absolute frequencies, percentages and ratios. Analysis were performed using IBM SPSS Statistics for Windows, version 21.0 (Chicago, ILL).

## Results

A total of 301 patients were evaluated in the pediatric critical care services under consideration. The total number of prescription drugs was 2347 with an average of 7.8 prescriptions per order; the highest being in the PICU (15.3 prescriptions per order). The general characteristics of the analyzed medication orders are shown in Table 1.

**Table 1:** Characteristics of medication orders in the pediatric critical care services

Pediatric Service	Orders, n	Prescriptions (drugs), n	Prescriptions/order (P/O)	Type of medication order, n (%)			Illegible medication orders^a^, n (%)	Amendments, n (%)		
				Hand-written	Digital	Combined^b^		Total	Legible	Illegible
PEC	88	715	8.1	0	27	61	7 (7.9)	8 (9.1)	7	1
PICU	66	1013	15.3	0	17	49	8 (12.1)	12 (17.6)	11	1
NICU	68	332	4.9	0	35	33	7 (10.2)	12 (17.6)	11	1
NIMCU	79	287	3.6	1	49	29	3 (3.8)	3 (3.8)	3	0
**TOTAL**	301	2347	7.8	1 (0.4)	128 (42.5)	172 (57.1)	25 (8.3)	35 (11.6)	32	3

*D* Drug, *I* Indication, *NICU* Neonatal Intensive Care Unit, *NIMCU* Neonatal Intermediate Care Unit, *PEC* Pediatric Emergency Care, *PICU* Pediatric Intensive Care Unit, *P* Prescription

^a^ Illegible medication orders would require a clarification from the attending physician to follow the therapeutic indication

^b^ Combined medication orders (handwritten + digital)

Among the 2347 prescriptions, a total of 1252 potential MEs (53.3%) and 379 clinically relevant MEs (16.1%) were identified. The pediatric services with the highest number of MEs were PICU (190) followed by PEC (139). Likewise, the PICU and the PEC had the highest clinically relevant prescription error rate (18.8 and 19.4% clinically relevant errors per prescription, respectively) and ME/patient index (1.6 and 2.9 clinically relevant errors per patient, respectively). The main findings are described in Table 2.

**Table 2:** Characteristics of MEs in the pediatric critical care services according to the updated classification by the Ruíz-Jarabo 2000 working group

Variables	Hospital pediatric critical care services					
	PEC	PICU	NICU	NIMCU	Total	
					n	%
Total potential medication errors, n (%)	358 (50.1)	643 (63.5)	141 (42.5)	110 (38.3)	1252	53.3
Total clinically relevant medication errors^b^, n	139	190	31	19	379	30.3
Clinically relevant Prescription Error Rate^b^ %	19.4	18.8	9.3	6.6	16.1	
Clinically relevant ME/patient Index	1.6	2.9	0.5	0.2	–	
Types of Medication Errors^a^, n (%)						
-Wrong speed rate of administration^b^	45	102	31	14	192	15.3
-Abbreviations	258	432	85	83	858	68.5
*Clinically relevant*^*b*^	65	42	0	5	112	13.1
*Not clinically relevant*	193	390	85	78	746	86.9
-Spelling error	26	63	25	13	127	10.1
-Misinterpretation/mistaken drug units^b^	29	46	0	0	75	6.0
Patients with ideal medication orders, n (%)	6 (6.8)	0 (0.0)	12 (17.6)	26 (32.9)	44	14.6
Prescriptions with ideal drugs, n (%)	17 (2.4)	146 (14.4)	45 (13.5)	66 (23.0)	274	11.7

*ME* Medication Error, *NICU* Neonatal Intensive Care Unit, *NIMCU* Neonatal Intermediate Special Care Unit, *PEC* Pediatric Emergency Care, *PICU* Pediatric Intensive Care Unit

^a^According to the Institute for Safe Medication Practices (ISMP) Spain (12); ^b^ The clinically relevant abbreviations were KCL (Potassium Chloride) and PHT (Phenytoin)

The potential clinical implications of MEs found in the critical care services including the mild or severe implications of the prescribed drugs are described in Table 3.

**Table 3:** Main drugs related to the most frequent medication errors in the pediatric critical care services (PCCS)

PCCS	Types of Medication Errors	n (%)	Drug related in MEs, n	Clinical Relevance or Possible clinical implication^*^	
				Mild	Severe
**PEC**	Wrong speed rate of administration	45 (11.7)	Omeprazole, 40	Abdominal pain, Nausea	Respiratory system reactions
			Ampicillin, 2	Phlebitis, Injection site pain	Morbilliform rash or Serious allergic reactions
			Metoclopramide, 1	Diarrhea	Acute dystonic reaction
			Amikacin, 1	Injection site pain	Hearing loss
			Ceftriaxone, 1	Rash, Injection site pain	Hematological disorders
	Abbreviations	258 (67.2)	Glucose solution, 98	Phlebitis	Hyperosmolar syndrome
			Potassium chloride, 53	Injection site pain, Phlebitis	Cardiac arrhythmias
			Saline solution, 28	Phlebitis	Hypervolemia
			Phenytoin, 12	Injection site pain, Rash	Cardiac rhythm disturbances
			Others, 67		
	Total No. of MEs	384			
**PICU**	Wrong speed rate of administration	102 (15.8)	Omeprazole, 59	Abdominal pain, Nausea	Respiratory system reactions
			Metoclopramide, 43	Diarrhea	Acute dystonic reaction
	Spelling error	63 (9.8)	Fentanyl, 38	Headache, Nausea, Vomiting	Loss of consciousness & Tachycardia
			Sufentanil, 15	Headache, Nausea, Vomiting	Loss of consciousness & Tachycardia
			Furosemide, 8	Electrolyte disorder	Cardiac rhythm disturbances
			Others, 2		
	Misinterpretation/mistaken drug units	46 (7.2)	Nystatin, 17	Oral irritation, Sensitization	Stevens-Johnson syndrome
			Albuterol, 15	Throat irritation, Nausea	Cardiac rhythm disturbances
			Ipratropium bromide / Albuterol, 12	Throat irritation, Nausea	Cardiac rhythm disturbances
			Epinephrine, 2	Dizziness, Sweating	Cardiac rhythm disturbances
	Abbreviations	432 (67.2)	Glucose solution, 174	Phlebitis	Hyperosmolar syndrome
			Potassium chloride, 25	Injection site pain, Phlebitis	Cardiac arrhythmias
			Saline solution, 66	Phlebitis	Hypervolemia
			Phenytoin, 17	Injection site pain, Rash	Cardiac rhythm disturbances
			Others, 150		
	Total of ME	643			
**NICU**	Wrong speed rate of administration	31 (22)	Omeprazole, 30	Abdominal pain, Nausea	Respiratory system reactions
			Metoclopramide, 1	Diarrhea, Hypotension	Acute dystonic reaction
	Spelling error	25 (17.7)	Fentanyl, 21	Headache, Nausea, Vomiting	Loss of consciousness & Tachycardia
			Furosemide, 2	Electrolyte disorder	Cardiac rhythm disturbances
			Others, 2		
	Abbreviations	85 (60.3)	Glucose solution, 55	Phlebitis	Hyperosmolar syndrome
			Saline solution, 9	Phlebitis	Hypervolemia
			Multivitamin, 4	Stomach discomfort	Hypervitaminosis
			Others, 17		
	Total No. of MEs	141			
**NIMCU**	Wrong speed rate of administration	14 (12.7)	Omeprazole, 12	Abdominal pain, Nausea	Respiratory system reactions
			Vancomycin, 1	Nausea	Hypokalemia & Red man Syndrome
			Amikacin, 1	Injection site pain	Hearing loss
	Spelling error	13 (11.8)	Fentanyl, 6	Headache, Nausea, Vomiting	Loss of consciousness & Tachycardia
			Furosemide, 3	Electrolyte disorder	Cardiac rhythm disturbances
			Others, 4		
	Abbreviations	83 (75.5)	Glucose solution, 37	Phlebitis	Hyperosmolar syndrome
			Potassium chloride, 5	Injection site pain, Phlebitis	Cardiac arrhythmias
			Saline solution, 9	Phlebitis	Hypervolemia
			Multivitamin, 4	Stomach discomfort	Hypervitaminosis
			Others, 28		
	Total No. of MEs	110			

*The clinical implications were consulted at https://www.drugs.com/sfx/ [25]

*HPMC* Hydroxypropyl methylcellulose, *ME* Medication Error, *NICU* Neonatal Intensive Care Unit, *NIMCU* Neonatal Intermediate Special Care Unit, *PCCS* Pediatric critical care services, *PEC* Pediatric Emergency Care, *PICU* Pediatric Intensive Care Unit

## Discussion

Medication errors occur frequently in pediatric critical care settings affecting patient safety and quality of healthcare [15, 26, 27]. According to our knowledge, this is the first study reporting pediatric MEs in emergency and intensive care services in Mexico. The higher frequency of MEs could be attributed to the fact that the study was conducted in a teaching hospital with limited full-time human resources and the lack of an electronic system for medication orders. On the other hand, the percentage of MEs in other countries, such as the USA [28] and Spain [29] have reported lower values ranging from 5 to 10%. This low frequency could reflect their rigorous health systems with electronic medical record use, as well as hospital pharmacy services, which are directly involved in the improvement of the medication process. Our study reports an occurrence and frequency of MEs that may also occur in other hospitals in Latin America, highlighting that improvements are still needed in pediatric critical care services.

Initially, 1252 MEs were detected representing 53.3%, however, after a thorough analysis, only clinically relevant MEs (379) that could cause harm to the patient were considered and this percentage was reduced to 16.1%. To obtain this percentage, abbreviations of glucose 5% and saline solution 0.9% (GS5%, SS0.9%, or SS, respectively) were discarded and only those abbreviations related to concentrated electrolytes and anticonvulsants, and the infusion rates or incorrect units of the registered drugs, were considered. These MEs are clinically relevant because according to the literature and clinical practice, patients with these errors are susceptible to mild skin reactions, such as phlebitis, rash, pain at the administration site and major complications that include cardiac rhythm disturbances, respiratory system reactions, and hypervolemia, among others [30].

The PICU and PEC areas had the highest number of MEs. This finding could be attributed, the high-stake and stressful situations that arise in these units, such as in resuscitations, where physicians must make immediate decisions in order to secure the well-being of the patients [31].

One study performed in the pediatric intensive care unit of a Spanish hospital found that 59% of all MEs were caused by some illegible element compared with 25% of our results for the same pediatric service [32]. Nevertheless, abbreviations were also the leading cause of MEs in our study (68.5%), however, only 13.1% were considered clinically relevant.

It is important to highlight that even though our study shows a high frequency of medication errors, most specialized hospitals in Mexico are teaching hospitals, which represents a greater probability of finding MEs, particularly in critical care services. Furthermore, when comparing our results with other studies performed in Mexico, three studies stand out, one by Villegas F [33] where the percentage of MEs was 8.9%; however, this study was conducted in a private and certified hospital, requiring specialized trained pharmacists. Another study performed in a public teaching hospital by del Rey-Pineda E. et al., reported a prescription error rate of 1.7% [34], which is very similar to PEC, and higher in two of the studied services (NICU and NIMCU). In the PICU however, the prescription error rate was almost twice as high compared to our results. Lastly, a review of prospective studies on medication prescription errors in Mexico conducted by Núñez-Sánchez A. et al., found an ME rate between 1.5 to 34% [35]. These findings demonstrate the wide variability of prescription rates in private vs public Mexican hospitals. In other countries with similar healthcare conditions to Latin America, one Brazilian study found a ME rate of 7.4% and Colombian study found a ME rate of 97.3% [36] with a follow-up publication estimating that 67.2% of these errors reach the patient [37, 38].

The importance of this study demonstrates the need to include trained pharmacists to the healthcare team in Mexican public hospitals; to validate prescriptions, to provide prescription training to health care providers, and most importantly, to enhance drug safety. Literature has demonstrated that clinical pharmacists in intensive care units play a key role in the prevention of MEs and adverse drug events [39]. Furthermore, the impact that a clinical pharmacist has on improving patient safety has also been reported in a tertiary level teaching hospital [40], where clinical pharmacists are an integral part of the medical staff, and most importantly, the specialized pediatric pharmacist is considered the best fit for the 24-h critical care needed.

In neonatal or premature critical care areas, the main ME was related to the speed of administration, similar to other reports [13, 14]. Also, a frequent ME was the use of abbreviations that could cause a misinterpretation of the medication order or a delay in the administration. It should be noted that patients in these areas are usually in critical states and neonates or premature infants do not have the ability to verbally express pain or physical discomfort, which could imply an overmedication when treating these symptoms. In addition, it has been reported that neonates are particularly vulnerable to the consequences of MEs as they are smaller in size and fragile, generally with immature organ systems. Therefore, the timely detection and prevention of MEs in neonates is particularly important given that neonates and premature infants are at high risk for error [9, 41, 42], with even minor errors possibly leading to short and long-term norm [26].

Of all the critical care areas evaluated, NIMCU presented the highest percentage of ideal medications for the specific needs of each patient, while PICU obtained the lowest percentage. This may be because the PICU service had a P/O numerically higher than NIMCU [15.3 vs. 3.6]. This P/O value means that PICU patients have a greater quantity of prescribed drugs (polypharmacy) compared to NIMCU patients, and therefore, an increased risk of MEs, drug interactions and adverse drug reactions. One way to confirm this risk was through the clinically relevant ME/patient index, where a higher index was observed in PICU compared to NIMCU (2.9 vs. 0.2), which is consistent with a higher number of MEs. These findings could reflect similar working environments for each pediatric critical care service in Mexican or in other Latin American teaching hospitals.

Limitations of this study include its cross-sectional design since it was not possible to determine the real consequences of MEs detected in these patients. Also, only the prescription phase was evaluated in this study and not the rest of the medication use process, such as the dispensation and administration stages, or sub-classifications (prescribed PRN vs scheduled or resuscitation situations), and disease severity in patients. Lastly, the direct observation method was time-consuming [27] since the pharmacist needed to attend each service to perform this task, limiting even further the number of detected MEs. Nonetheless, this study exposes the need to improve the quality of the prescription process in pediatric critical care patients, and in this regard, a long-term multicentric study is suggested.

Several proposals to mitigate MEs would be the creation of prescription programs and constant training for healthcare professionals, the implementation of pharmaceutical care to validate medication orders (prescribed doses, routes and interactions, among others.

## Conclusion

Medication errors were frequent in all pediatric critical care services during the prescription stage due to the complex and stressful nature of the critical care setting. The need to establish reporting systems for the prevention of MEs through the participation of a pharmacist can significantly reduce their incidence due to an early and timely detection and thus, improve patient safety for the pediatric population in critical care services.

The findings from this study provide the framework for future longitudinal studies that include the intervention of pharmacists to minimize MEs, not only in the prescription stage, but in the other stages of the medication use process before they can reach or cause harm to patients.

## Data Availability

The datasets generated during and/or analyzed during the current study are not publicly available due to previous confidentiality agreements with the Hospital’s Ethics and Research Committees but are available from the corresponding author on reasonable request.

## Abbreviations

ME: Medication errors
NICU: Neonatal Intensive Care Unit
NIMCU: Neonatal Intermediate Care Unit
PICU: Pediatric Intensive Care Unit
PEC: Pediatric Emergency Care
NCC MERP: Coordinating Council for Medication Error Reporting and Prevention
P/O: Prescription/order
